# Alpha‐Linolenic Acid and Mortality Among Adults With Type 2 Diabetes: Findings From Two National Cohorts

**DOI:** 10.1111/1753-0407.70110

**Published:** 2025-06-22

**Authors:** Boyang Chen, Qi Wu, Sibo Liu, Hongkun Di, Wen Hu, Tianzhu Qin, Yushuang Wang, Rong Chen, Han Wang, Ying Chen, Xiang Cheng, Jiawei Yin, Liegang Liu, Zhilei Shan

**Affiliations:** ^1^ Department of Nutrition and Food Hygiene, Hubei Key Laboratory of Food Nutrition and Safety, School of Public Health Tongji Medical College, Huazhong University of Science and Technology Wuhan China; ^2^ Ministry of Education Key Lab of Environment and Health, School of Public Health, Tongji Medical College Huazhong University of Science and Technology Wuhan China; ^3^ Institute of Nutrition and Food Hygiene Beijing Changping District Center for Disease Control and Prevention Beijing China; ^4^ Ministry of Education Key Laboratory of Metabolism and Molecular Medicine, Department of Endocrinology and Metabolism Zhongshan Hospital, Fudan University Shanghai China; ^5^ Department of Cardiology Tongji Medical College Union Hospital, Huazhong University of Science and Technology Wuhan China; ^6^ Department of Nutrition and Food Hygiene, School of Public Health Chongqing Medical University Chongqing China; ^7^ Nutrition Innovation Platform‐Sichuan and Chongqing, School of Public Health Chongqing Medical University Chongqing China

**Keywords:** alpha‐linolenic acid, cardiovascular mortality, cohort analysis, mortality, type 2 diabetes

## Abstract

**Background:**

Dietary alpha‐linolenic acid (ALA) regulates lipid metabolism and insulin sensitivity, but few studies have investigated the association between ALA and the risk of mortality among adults with type 2 diabetes (T2D). This study examines whether increasing dietary ALA intake contributes to the long‐term survival of adults with T2D.

**Methods:**

This cohort study included 9603 participants with T2D, including 7953 adults from the National Health and Nutrition Examination Survey (NHANES; 1999–2018) and 1650 adults from the China Health and Nutrition Survey (CHNS; 1997–2011). Dietary information was collected through 24‐h dietary recalls. Cox proportional hazards regression was employed to estimate hazard ratios (HRs) and 95% CIs for mortality from all‐cause and cardiovascular disease (CVD).

**Results:**

During 75 535 person‐years of follow‐up, a total of 2468 deaths were documented. After multivariate adjustment, the pooled HRs (95% CIs) of all‐cause mortality were 1.00, 0.87 (0.76–0.99), and 0.79 (0.67–0.94) across tertiles of ALA (*p*_trend_ = 0.01). There was a linear inverse relationship between ALA intake and all‐cause mortality, demonstrating a 9% (HR: 0.91;95% CI: 0.85_0.97) lower risk of all‐cause mortality with each 1 g/day increase of dietary ALA intake in the pooled analysis (*p*_nonlinear_ > 0.05). In addition, ALA intake was inversely associated with CVD mortality, and HR comparing the highest with the lowest tertile was 0.68 (0.50–0.91; *p*_trend_ = 0.01). Consistent results were observed in both the stratified and sensitivity analyses.

**Conclusions:**

Higher dietary ALA intake was associated with a lower risk of all‐cause and CVD mortality among adults with T2D.

## Graphical abstract

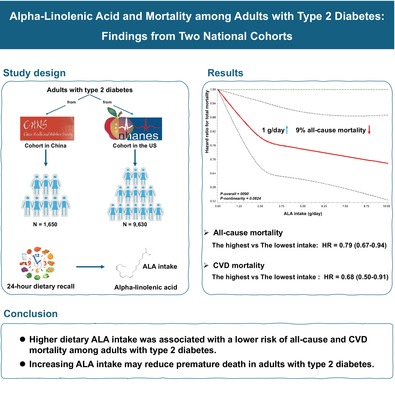

**Summary**

- Each 1 g/day increment in ALA intake was linked to a 9% reduced risk of all‐cause mortality in adults with T2D.
- Increased ALA intake was associated with improvements in certain cardiometabolic biomarkers, such as total cholesterol and LDL‐C.
- Increasing intake of ALA may reduce premature death in adults with T2D.

**Abbreviations**
ALA: Alpha‐linolenic acid
BMI: Body mass index
CHD: Coronary heart disease
CHNS: China Health and Nutrition Survey
CI: Confidence interval
CRP: C‐reactive protein
CVD: Cardiovascular disease
DHA: Docosahexaenoic acid
DPA: Docosapentaenoic acid
EPA: Eicosapentaenoic acid
HbA1c: Glycosylated hemoglobin A1c
HDL‐C: High‐density lipoprotein cholesterol
HOMA‐2IR: Homoeostasis model assessment 2 estimates of insulin resistance
HPFS: Health Professionals Follow‐Up Study
HR: Hazard ratio
LDL‐C: Low‐density lipoprotein cholesterol
MUFAs: Monounsaturated fatty acids
NHANES: National Health and Nutrition Examination Survey
NHS: Nurses' Health Study
OGTT: Oral glucose tolerance test
PUFAs: Polyunsaturated fatty acids
RCS: Restricted cubic spline
T2D: Type 2 diabetes
TC: Total cholesterol
TG: triglyceride

## 1 Introduction

Type 2 diabetes (T2D) is a global chronic health issue, increasing a substantial social and economic burden on healthcare systems and imposing life‐threatening implications [1]. Evidence shows that individuals with T2D have a 2–3 times higher risk of cardiovascular events, which are the primary cause of mortality in this population, compared to those without T2D [2]. Accumulating evidence indicates that dietary omega‐3 fatty acids play a crucial role in mitigating the progression of diabetes and its complications [3, 4, 5, 6].

Alpha‐linolenic acid (ALA; C18:3), a plant‐derived omega‐3 fatty acid, cannot be synthesized endogenously in vivo and must be obtained from dietary sources. Flaxseed, walnuts, rapeseed, and their respective oil products are the primary dietary sources of ALA [7]. Many studies have demonstrated that dietary ALA is involved in regulating the inflammation response, lipid metabolism, and insulin sensitivity [8, 9, 10, 11]. A recent meta‐analysis of cohort studies showed inverse associations of ALA intake with mortality from all causes, cardiovascular disease (CVD), and coronary heart disease (CHD) in the general population [12]. Another meta‐analysis of randomized controlled trials has also indicated the beneficial effects of ALA supplementation in reducing the risk of CVD events, fatal CHD, and arrhythmia [13]. However, evidence is limited for adults with T2D. Currently, the existing evidence, based on the Nurses' Health Study (NHS) and the Health Professionals Follow‐up Study (HPFS), has found a marginally reverse association of ALA intake in comparison with saturated fatty acids (SFAs) with all‐cause mortality but no significant association with CVD mortality among populations with T2D [14]. Notably, these findings derived from health professionals may lack generalizability to broader populations. The current dietary advice recommends an ALA intake of 0.6% energy intake for the Chinese general population, which is approximately 1.4–1.7 g/day for individuals engaged in moderate‐intensity physical activity, while for Americans, the recommendation is about 1.1 g/day for women and 1.6 g/day for men [15]. However, the applicability of these dietary recommendations to adults with T2D is uncertain owing to their unique abnormal lipid and glucose metabolism.

Therefore, this study was conducted to investigate the associations of dietary ALA intake with all‐cause and CVD mortality among adults with T2D in the National Health and Nutrition Examination Survey (NHANES) and the China Health and Nutrition Survey (CHNS), aiming to inform evidence‐based dietary recommendations for this high‐risk group.

## 2 Materials and Methods

### 2.1 Study Population

Our study was based on NHANES for the US population and CHNS for the Chinese population, respectively. NHANES is a large‐scale survey designed to assess the health and nutritional status, utilizing representative samples of the US civilian population. Meanwhile, CHNS is an ongoing household‐based longitudinal cohort study in China, employing a multistage random‐cluster method to collect a sample of over 30 000 individuals from 9 provinces. The data of NHANES were reviewed and approved by the National Center for Health Statistics Research Ethics Review Board, and informed consent was obtained from all participants. Detailed methodological descriptions of both surveys have been published previously [16, 17].

In this study, individuals with T2D from continuous cycles of NHANES (1999–2018) and CHNS (1997–2011) were included. Individuals were diagnosed with T2D if they met at least one of the following criteria: (1) a self‐reported diagnosis of diabetes by a specialist; (2) fasting blood glucose ≥ 7.0 mmol/L, hemoglobin A1c (HbA1c) ≥ 6.5%, or 2‐h oral glucose tolerance test (2 h‐OGTT) ≥ 11.1 mmol/L; (3) using hypoglycemic medicine or taking insulin. For the current analysis, participants with T2D who self‐reported as pregnant, reported extreme energy intake (total energy < 500 or > 3500 kcal/day for women; < 800 or > 4200 kcal/day for men), had missing or duplicated dietary records, or had missing or illogical information about death were excluded. Overall, 9603 participants aged 20 years and above were included in analyses (*n* = 7953 in NHANES 1999–2018 and *n* = 1650 in CHNS 1997–2011).

### 2.2 Measurement of Dietary Alpha‐Linolenic Acid Intake

The nutritional assessment in NHANES (1999–2018) involved a 24‐h dietary recall interview conducted in person by trained interviewers in a private room in the Mobile Examination Center (MEC). In the first two cycles, a single dietary interview was conducted on a randomly chosen day. From 2003 onward, a second interview was conducted by telephone 3 to 10 days after the initial interview. To represent long‐term dietary habits and minimize individual differences, the mean dietary information was calculated in the cycles from 2003 to 2018. For participants enrolled in 1999–2002 or lacking a second dietary interview, only one 24‐h recall was used. Daily aggregates of food energy and macronutrient components (fats, protein, and carbohydrates) from all foods were calculated using the United States Department of Agriculture's (USDA) Food and Nutrient Database for Dietary Studies (FNDDS), based on cycle‐specific versions [18]. In CHNS (1997–2011), dietary intake was assessed using a three‐day consecutive 24‐h dietary recall and household food checklist to estimate the typical consumption of participants. Dietary ALA and other nutrient values were calculated based on updated versions of the Chinese Food Composition Table (FCT) for each round [19].

### 2.3 Ascertainment of Mortality

The follow‐up period was calculated from baseline to the date of death or until 31 December 2019 for NHANES, and until 31 December 2015 for CHNS, whichever occurred first. Information on all‐cause mortality and specific mortality was ascertained by linking to the National Death Index (NDI) through 31 December 2019 for NHANES. The cause of death was determined using the 10th revision of the International Statistical Classification of Diseases and Related Health Problems (ICD‐10) guidelines. Death due to CVD was defined as ICD‐10 codes I00–I09, I11, I13, I20–I51, or I60–I69. In CHNS, information on mortality was ascertained according to the report collected from household members in each survey. If the death record of a participant was reported multiple times, we initially used the data from the first report. As cause‐specific death information was only reported in the 1991 survey, there was no information available on cause‐specific mortality in CHNS.

### 2.4 Assessment of Covariates

Several possible confounding variables related to demographics, social economics, health‐related behavior, and history of diseases previously associated with mortality [6, 14, 20], were collected by standardized questionnaires or physical examinations at baseline, including age, sex, race/ethnicity, education levels, economic status, drinking and smoking habits, leisure‐time physical activity, duration and medication for diabetes, as well as a history of hypertension, hypercholesterolemia, CVD, and cancer. Additionally, the family history of diabetes and CVD was also documented.

BMI was determined by dividing the weight in kilograms by the square of the height in meters (kg/m^2^) and then categorized into 4 groups for NHANES (< 18.5, 18.5–24.9, 25.0–29.9, or ≥ 30.0) and for CHNS (< 18.5, 18.5–23.9, 24.0–27.9, or ≥ 28.0) [21]. Education levels were grouped as less than high school graduate, high school graduate or General Equivalent Diploma (GED), and college or above. Economic status was classified based on the ratio of family income to poverty level for NHANES or household per capita annual income for CHNS. Drinkers were categorized as non‐drinker, ever‐drinker, low and moderate drinker, and heavy drinker based on consumption levels and frequency. For smoking status, individuals were divided into never smoker, ever smoker, and current smoker with further subdivision based on the number of cigarettes smoked per day. Leisure‐time physical activity was defined as inactive group, insufficiently active group, and active group. The duration of diabetes was categorized into < 3, 3 to 10, and > 10 years. Individuals with hypertension/hypercholesterolemia should have self‐reported high blood pressure/cholesterol levels or be taking prescription or prescribed medicine for treatment. Suffering from CVDs was defined as self‐reported suffering from at least one of the following diseases: congestive heart failure, coronary heart disease, angina/angina pectoris, heart attack, and stroke. Participants in NHANES were classified into two groups based on whether they reported taking ALA supplements or not. They were also asked about their ALA supplement usage in the past 30 days, including the amount and duration of supplementation.

In addition, cardiometabolic markers such as fasting glucose, fasting insulin, HbA1c, triglyceride (TG), total cholesterol (TC), high‐density lipoprotein cholesterol (HDL‐C), low‐density lipoprotein cholesterol (LDL‐C), and C‐reactive protein (CRP) were measured at recruitment. The insulin resistance (HOMA2‐IR) was determined based on fasting blood glucose (mmol/L) and insulin (pmol/L), calculated using the HOMA calculator developed by the University of Oxford, UK [22].

### 2.5 Statistical Analysis

Participants were categorized into three groups based on the tertiles of dietary ALA intake. Baseline characteristics were presented as weighted means (standard errors, SE) or proportions. *P* values for differences across ALA tertiles were estimated using ANOVA for continuous variables or chi‐squared tests for categorical variables. The generalized linear model was used to examine the associations of ALA intake with cardiometabolic biomarkers at baseline, including fasting glucose, insulin, HOMA2‐IR, HbA1c, triglyceride, TC, HDL‐C, LDL‐C, and CRP.

Multivariable Cox proportional hazards regression models were used to calculate hazard ratios (HRs) and 95% confidence intervals (CIs) to examine the associations between dietary ALA intake and all‐cause mortality or CVD mortality (only for NHANES) among adults with T2D. In model 1, we adjusted for age, sex, and race/ethnicity. In model 2, we further adjusted for BMI, education level, economic status, drinking status, smoking status, leisure‐time physical activity, history of hypertension, history of hypercholesterolemia, history of cancer, history of CVDs, family history of CVDs (only for NHANES), family history of diabetes (only for NHANES), duration of diabetes, diabetes medication use, ALA supplement (only for NHANES), and total energy intake. Linear trend tests were conducted using the median of each tertile of ALA intake as a continuous variable. Missing values were imputed with missing indicators for categorical variables. Analyses were conducted separately for each cohort, followed by a fixed‐effects meta‐analysis using the inverse‐variance weighted method to combine the results of both cohorts. Heterogeneity was evaluated using the *I* ^2^ statistic.

Restricted cubic spline (RCS) regression with three knots (at the 25th, 50th, and 75th percentiles) was used to explore the dose–response relationship between ALA intake and mortality. Nonlinearity was assessed using likelihood ratio tests. Stratified analyses were conducted by median age of individuals in two cohorts (< 60, ≥ 60 years), sex (male or female), race/ethnicity (NHANES: non‐Hispanic white, other; CHNS: Han, the minority), education level (less than high school graduate, or high school graduate or above), BMI (< median or ≥ median, NHANES: < 30 or ≥ 30; CHNS: < 25 or ≥ 25), economic status (NHANES: < 3.0, or ≥ 3.0; CHNS: ¥, < 5000, or ≥ 5000), drinking status (nondrinker or drinker), smoking status (never, ever or current), leisure‐time physical activity (inactive or active), medication for diabetes (none or pills and/or insulin), diabetes duration (< 3 or ≥ 3 years), hypertension and/or hypercholesterolemia (yes or no), and CVD and/or cancer (yes or no). Further stratified analyses were conducted by median intake of marine n‐3 PUFA for all‐cause mortality and CVD mortality. The interaction effects were estimated by likelihood ratio tests. To ensure the robustness of the results, several sensitivity analyses were performed. First, we excluded adults with a history of CVD or cancer to mitigate the potential influences of fatal diseases on our results. Second, participants who died within the first 4 years of the follow‐up period were excluded to reduce the potential reverse causation bias. Third, participants from 1999 to 2002 in NHANES were excluded. Fourth, adults with extreme BMI (< 18.5 or > 40 kg/m^2^) were excluded. Fifth, dietary ALA units were converted to the percentage of energy intake. Sixth, multiple imputation was used to address missing values. Finally, the *E*‐value was calculated to estimate the robustness of the analysis of associations between ALA intake and mortality due to the unmeasured confounders [23]. Higher *E*‐value indicates stronger evidence supporting the observed associations. The analyses were conducted using SAS (version 9.4). All statistical tests were two‐sided, and *p* < 0.05 was considered to indicate statistical significance.

## 3 Results

### 3.1 Participant Characteristics

The final analysis included 9603 adults with T2D, comprising 7953 participants from NHANES and 1650 participants from CHNS. In NHANES, the mean (SE) age was 59.8 (0.3), with 4046 males weighted at 50.4%; in CHNS, the mean (SE) age was 59.9 (0.3), with 811 males weighted at 49.2%. The median (IQR) dietary ALA intake was 1.32 (0.87 to 1.94) g/day for American participants and 1.54 (0.54 to 2.84) g/day for Chinese participants. Table 1 shows the baseline characteristics of adults with T2D in NHANES and CHNS categorized by tertiles of ALA intake. At baseline, adults with T2D with higher ALA intake tended to be younger men, more likely to have higher education and better quality of living, and to consume higher calories, carbohydrates, and various fatty acids, including SFAs, monounsaturated fatty acids (MUFAs), and polyunsaturated fatty acids (PUFAs) in both cohorts. In NHANES, individuals with higher ALA intake were more likely to be drinkers, overweight/obese, and suffer from hypercholesterolemia, but less likely to have a family history or suffer from CVDs. In CHNS, participants with higher ALA intake tended to be smokers and were more likely to have a short duration of diabetes.

**TABLE 1:** Characteristics of participants with type 2 diabetes according to tertiles of alpha‐linolenic acid intake.

Characteristics^a^	Total	Tertiles of ALA intake			*p* ^b^
		T1	T2	T3	
NHANES					
Median ALA intake (IQR), g/day	1.32 (0.87–1.94)	0.68 (0.51–0.83)	1.25 (1.11–1.41)	2.17 (1.85–2.81)	
No. of participants	7953	2650	2651	2652	
Age, years	59.8 (0.3)	60.2 (0.5)	60.4 (0.4)	58.9 (0.4)	0.008
Male	4046 (50.4)	1048 (38.2)	1378 (49.9)	1620 (60.8)	< 0.0001
Non‐Hispanic White	2961 (62.2)	890 (57.4)	1001 (61.9)	1070 (66.4)	< 0.0001
College or above	3188 (48.3)	807 (39.5)	1069 (47.8)	1312 (56.0)	< 0.0001
Ratio of family income to poverty level					
≤ 1.0	1671 (15.7)	678 (20.9)	558 (15.6)	435 (11.5)	< 0.0001
1.0–3.0	3377 (38.6)	1163 (40.9)	1143 (38.8)	1071 (36.4)	
> 3.0	2163 (38.0)	530 (28.7)	699 (37.6)	934 (46.2)	
BMI, kg/m^2^					
< 18.5	31 (0.4)	13 (0.4)	11 (0.5)	7 (0.2)	0.006
18.5–24.9	1039 (12.1)	384 (14.8)	354 (12.0)	301 (9.9)	
25.0–29.9	2337 (26.7)	779 (26.1)	774 (26.9)	784 (27.0)	
≥ 30.0	4336 (58.7)	1401 (56.2)	1426 (58.2)	1509 (61.2)	
Current drinker	3395 (48.7)	921 (41.7)	1144 (49.2)	1330 (54.2)	< 0.0001
Current smoker	3034 (38.9)	965 (36.4)	990 (38.7)	1079 (41.1)	0.210
Leisure‐time physical activity					
Inactive	3732 (44.1)	1214 (42.8)	1225 (43.9)	1293 (45.4)	< 0.0001
Active	2901 (40.7)	857 (37.0)	991 (41.3)	1053 (43.1)	
ALA supplement	46 (0.8)	8 (0.4)	13 (0.6)	25 (1.3)	0.080
Duration of diabetes, years					
< 3	3497 (45.6)	1133 (45.7)	1193 (46.4)	1171 (44.7)	0.790
3–10	1814 (23.0)	616 (22.8)	584 (22.5)	614 (23.6)	
> 10	2427 (28.5)	822 (28.7)	820 (28.6)	785 (28.2)	
Medication for diabetes					
None	1268 (17.9)	374 (16.6)	429 (19.2)	465 (17.8)	0.002
Only diabetes pills	3533 (42.9)	1189 (40.1)	1108 (40.6)	1236 (47.4)	
Only insulin	751 (9.6)	265 (11.4)	245 (8.7)	241 (8.8)	
Pills and insulin	812 (10.0)	268 (10.6)	283 (10.5)	261 (9.2)	
Family history of diabetes	5078 (63.5)	1669 (64.5)	1664 (61.8)	1745 (64.1)	0.350
Family history of cardiovascular diseases	1587 (21.9)	580 (24.8)	512 (20.2)	495 (21.0)	0.020
Hypertension	5149 (63.8)	1752 (62.7)	1710 (64.5)	1687 (64.0)	0.640
Hypercholesterolemia	4534 (59.1)	1474 (55.1)	1488 (58.6)	1572 (62.8)	0.002
Cardiovascular disease	2061 (25.1)	747 (27.2)	710 (25.9)	604 (22.7)	0.040
Cancer	1115 (15.9)	370 (15.5)	396 (17.0)	349 (15.2)	0.370
Total energy intake, kcal/day	1862.3 (12.7)	1336.3 (13.0)	1818 (15.8)	2336.6 (18.2)	< 0.0001
Nutrient intake, %energy/day					
Carbohydrates	48.5 (0.0)	52.2 (0.0)	48.4 (0.0)	44.9 (0.0)	< 0.0001
Protein	16.8 (0.0)	17.6 (0.0)	16.7 (0.0)	16.2 (0.0)	< 0.0001
Total fatty acids	34.6 (0.0)	30.1 (0.0)	34.7 (0.0)	38.8 (0.0)	< 0.0001
Saturated fatty acids	11.0 (0.0)	10.1 (0.0)	11.2 (0.0)	11.7 (0.0)	< 0.0001
Monounsaturated fatty acids	12.5 (0.0)	11.1 (0.0)	12.6 (0.0)	13.8 (0.0)	< 0.0001
Polyunsaturated fatty acids	7.9 (0.0)	5.9 (0.0)	7.7 (0.0)	10.0 (0.0)	< 0.0001
CHNS					
Median ALA intake (IQR), g/day	1.54 (0.54–2.84)	0.33 (0.18–0.54)	1.54 (1.15–1.93)	3.58 (2.84–4.80)	
No. of participants	1650	550	550	550	
Age, years	59.9 (0.3)	61.7 (0.5)	59.6 (0.5)	58.3 (0.5)	< 0.0001
Male	811 (49.2)	250 (45.5)	260 (47.3)	301 (54.7)	0.005
Han	1522 (92.2)	512 (93.1)	499 (90.7)	511 (92.9)	0.260
College or above	103 (6.2)	32 (5.8)	45 (8.2)	26 (4.7)	0.010
Household per capita annual income					
< 5,000	1108 (67.2)	393 (70.2)	379 (68.9)	343 (62.4)	0.030
5000–10 000	220 (13.3)	73 (13.3)	68 (12.4)	79 (14.4)	
≥ 10 000	322 (19.5)	91 (16.6)	103 (18.7)	128 (23.3)	
BMI, kg/m^2^					
< 18.5	138 (8.4)	54 (9.8)	44 (8.0)	40 (7.3)	0.470
18.5–23.9	597 (36.2)	190 (34.6)	207 (37.6)	200 (36.4)	
24.0–27.9	593 (35.9)	200 (36.4)	184 (33.5)	209 (38.0)	
≥ 28.0	322 (19.5)	106 (19.3)	115 (20.9)	101 (18.4)	
Current drinker	297 (18.0)	89 (16.2)	97 (17.6)	111 (20.2)	0.080
Current smoker	506 (30.7)	157 (28.5)	170 (30.9)	179 (32.5)	0.110
Leisure‐time physical activity					
Inactive	917 (55.6)	364 (66.2)	321 (58.4)	232 (42.2)	< 0.0001
Active	733 (45.4)	186 (33.8)	229 (41.6)	318 (57.8)	
Duration of diabetes, years					
< 3	1148 (69.6)	386 (70.2)	355 (64.6)	407 (74.0)	0.020
3–10	168 (10.2)	54 (9.8)	65 (11.8)	49 (8.9)	
> 10	334 (20.2)	110 (20.0)	130 (23.6)	94 (17.1)	
Medication for diabetes					
None	686 (41.6)	246 (44.7)	201 (36.6)	239 (43.5)	0.070
Only diabetes pills	847 (51.3)	264 (48)	311 (56.6)	272 (49.5)	
Only insulin	36 (2.2)	12 (2.2)	9 (1.6)	15 (2.7)	
Pills and insulin	81 (4.9)	28 (5.1)	29 (5.3)	24 (4.4)	
Hypertension	895 (54.2)	299 (54.4)	309 (56.2)	287 (52.2)	0.410
Hypercholesterolemia	395 (23.9)	132 (24.0)	128 (23.3)	135 (24.6)	0.880
Cardiovascular disease	375 (22.7)	133 (24.2)	132 (24.0)	110 (20.0)	0.170
Cancer	12 (0.7)	2 (0.4)	8 (1.5)	2 (0.4)	0.050
Total energy intake, kcal/day	1992.4 (16.0)	1769.1 (25.7)	1951 (26.5)	2257.2 (26.7)	< 0.0001
Nutrient intake, %energy/day					
Carbohydrates	55.2 (0.0)	57.7 (0.0)	57.1 (0.0)	50.6 (0.0)	< 0.0001
Protein	13.0 (0.0)	13.1 (0.0)	13.5 (0.0)	12.2 (0.0)	< 0.0001
Total fatty acids	33.3 (0.0)	30.6 (0.0)	30.69 (0.0)	38.5 (0.0)	< 0.0001
Saturated fatty acids	4.6 (0.0)	4.3 (0.0)	4.3 (0.0)	5.1 (0.0)	< 0.0001
Monounsaturated fatty acids	8.5 (0.0)	7.3 (0.0)	7.6 (0.0)	10.6 (0.0)	< 0.0001
Polyunsaturated fatty acids	8.2 (0.0)	5.9 (0.0)	7.4 (0.0)	11.3 (0.0)	< 0.0001

Abbreviations: ALA, alpha‐linolenic acid; CHNS, China Health and Nutrition Survey; IQR, inter‐quartile range; NHANES, National Health and Nutrition Examination Survey.

^a^ Data are presented as mean (SE) or *n* (%).

^b^ *P* values for differences in baseline characteristics were estimated by ANOVA or chi‐squared test.

### 3.2 Main Associations Between ALA and Risk of Mortality

**FIGURE 1:**
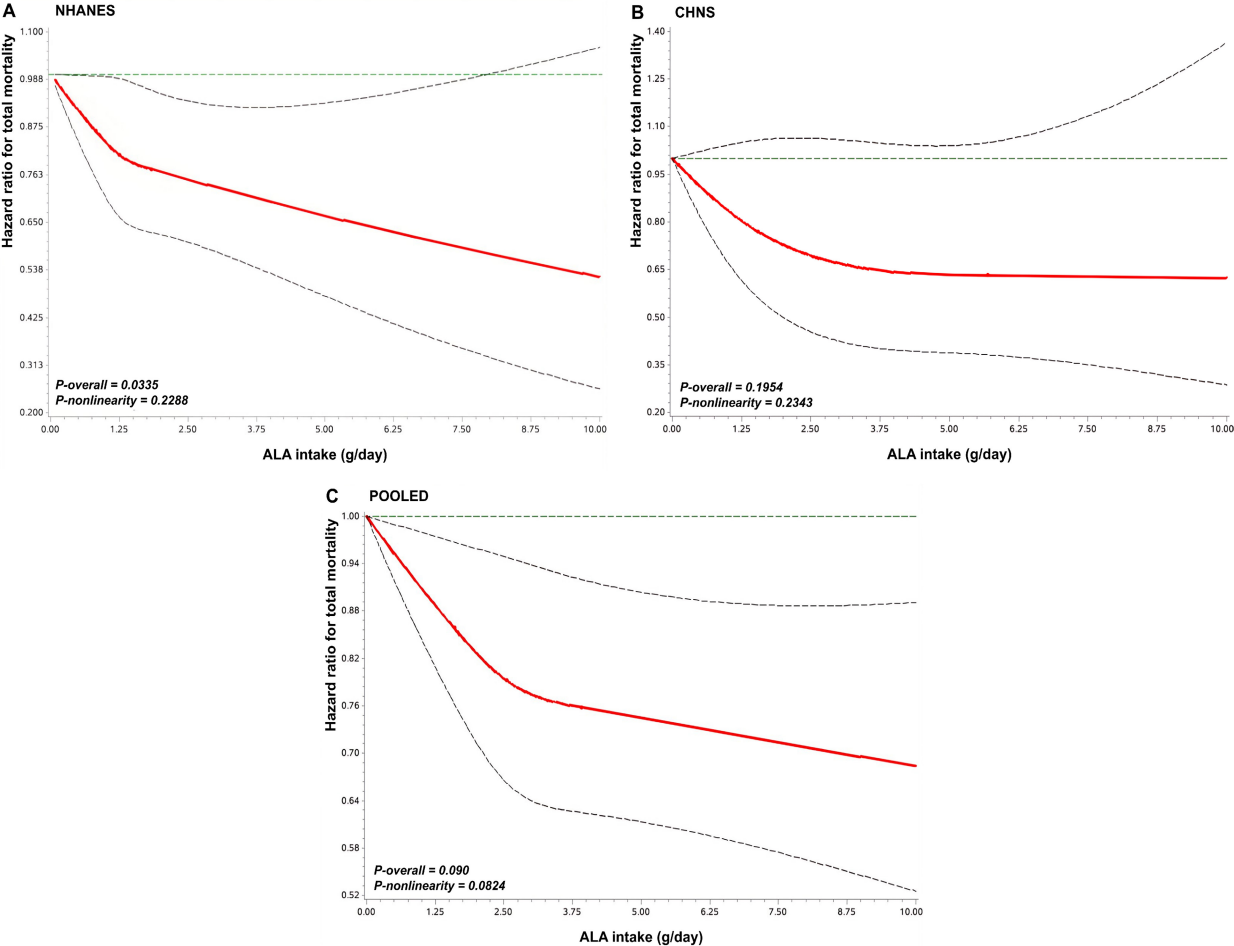
The dose–response relation between alpha‐linolenic acid intake and all‐cause mortality with restricted cubic spline regression model in NHANES (A), CHNS (B) and pooled (C). Abbreviations: ALA, alpha‐linolenic acid; CHNS, China Health and Nutrition Survey; NHANES, National Health and Nutrition Examination Survey. HRs were adjusted for age, sex, race/ethnicity, BMI, education level, economic status, drinking status, smoking status, leisure‐time physical activity, history of hypertension, history of hypercholesterolemia, history of cancer, history of cardiovascular diseases, family history of cardiovascular diseases, family history of diabetes, duration of diabetes, diabetes medication use, ALA supplement, and total energy intake.

During 63 153 person‐years of follow‐up in NHANES, 2301 deaths were documented, including 780 attributed to CVD. In CHNS, during 12 382 person‐years of follow‐up, 167 deaths were recorded. As depicted in Table 2, ALA intake was associated with a significantly reduced risk of all‐cause mortality after multivariate adjustment for demographics, health‐related behavior, history of diseases, diabetes duration, and diabetes medication use. Hazard ratios (HRs) across categories were 1.00, 0.89 (95% CI, 0.77–1.03), and 0.80 (0.66–0.96) for NHANES (*p*_trend_ = 0.02). A linearly reverse association was observed (*p*_nonlinear_ > 0.05, Figure 1). For CHNS, the HRs for all‐cause mortality were 1.00, 0.72 (0.49–1.06), and 0.75 (0.50–1.11) (*p*_trend_ = 0.19), showing no significant linear association. In the pooled analysis, the HRs were 1.00, 0.87 (0.76–0.99), and 0.79 (0.67–0.94) (*p*_trend_ = 0.01), with the linearly reverse association remaining significant (*p*_nonlinear_ > 0.05, Figure 1). For CVD mortality, compared with the lowest tertile, the HRs were 0.81 (0.65–1.01) for tertile 2 and 0.68 (0.50–0.91) for tertile 3 (*p*_trend_ = 0.01) in NHANES (Table S1), showing linear association (Figure S2). However, no further analysis was conducted for ALA and CVD mortality in CHNS because specific causes of death were not available.

**TABLE 2:** Associations of alpha‐linolenic acid intake with all‐cause mortality among adults with type 2 diabetes.

	Tertiles of ALA intake			Per 1 g/day increase	*p*_trend_
	T1 (lowest)	T2 (medium)	T3 (highest)		
NHANES					
ALA intake, g/day	0.68 (< 1.01)	1.25 (1.01–1.67)	2.17 (> 1.67)		
No. of deaths/person‐years	926/22 559	777/20 954	598/19 640		
Model 1^a^	1.00	0.85 (0.75, 0.97)	0.72 (0.62, 0.83)	0.84 (0.78, 0.91)	< 0.001
Model 2^b^	1.00	0.89 (0.77, 1.03)	0.80 (0.66, 0.96)	0.88 (0.80, 0.97)	0.02
CHNS					
ALA intake, g/day	0.33 (< 0.81)	1.54 (0.81–2.37)	3.58 (> 2.37)		
No. of deaths/person‐years	66/3727	50/4374	51/4281		
Model 1^a^	1.00	0.69 (0.48, 1.01)	0.76 (0.52, 1.09)	0.95 (0.87, 1.03)	0.19
Model 2^b^	1.00	0.72 (0.49, 1.06)	0.75 (0.50, 1.11)	0.94 (0.86, 1.03)	0.19
Pooled^c^					
Model 1^a^	1.00	0.83 (0.74, 0.94)	0.73 (0.63, 0.83)	0.89 (0.84, 0.94)	0.01
Model 2^b^	1.00	0.87 (0.76, 0.99)	0.79 (0.67, 0.94)	0.91 (0.85, 0.97)	0.01

Abbreviations: ALA, alpha‐linolenic acid; CHNS, China Health and Nutrition Survey; NHANES, National Health and Nutrition Examination Survey.

^a^ Adjusted for age, sex, and race/ethnicity.

^b^ Further adjusted for BMI, education level, economic status, drinking status, smoking status, leisure‐time physical activity, history of hypertension, history of hypercholesterolemia, history of cancer, history of cardiovascular diseases, family history of cardiovascular diseases, family history of diabetes, duration of diabetes, diabetes medication use, ALA supplement, and total energy intake.

^c^ Results for NHANES and CHNS from the multivariable model were combined using the fixed‐effects model.

In the subgroup analysis, consistent findings were observed and no significant interactions were found between ALA intake and the risk of all‐cause mortality (all *p*_interaction_ > 0.05, Table 3). As well as for marine n‐3 PUFA, although the result showed that dietary ALA had a stronger association with the risk of all‐cause and CVD mortality in the population with insufficient intake of marine n‐3 PUFA (< median), this interaction term was not statistically significant (*p*_interaction_ > 0.05, Tables 3 and S2). In the stratified analysis, the associations of ALA intake with all‐cause mortality persisted, even after excluding participants with CVD or cancer, those who died within the first 4 years since baseline, participants from 1999 to 2002 in NHANES, and participants with extreme BMI values. Furthermore, the conversion of ALA unit from grams to percentage of total energy intake or using multiple imputation to address missing values did not alter the primary results (Table S3). The results of the relation between dietary ALA and CVD mortality were consistent with the primary analysis (Table S4). Additionally, the *E*‐value for the point estimate and upper confidence bound for the association between ALA intake and all‐cause mortality was 1.63 and 1.26, respectively, and for CVD mortality was 2.30 and 1.43.

**TABLE 3:** Subgroup analyses of alpha‐linolenic acid intake and all‐cause mortality among adults with type 2 diabetes in NHANES 1999–2018 and CHNS 1997–2011.

	NHANES	*p*_interaction_	CHNS	*p*_interaction_	Pooled^a^	*p*_interaction_
Age, years						
< 60	0.85 (0.69, 1.04)	0.73	1.01 (0.83, 1.23)	0.46	0.93 (0.81, 1.07)	0.95
≥ 60	0.88 (0.78, 0.99)		0.93 (0.83, 1.03)		0.91 (0.84, 0.98)	
Sex						
Male	0.87 (0.76, 1.00)	0.78	0.96 (0.87, 1.07)	0.89	0.93 (0.85, 1.01)	0.27
Female	0.89 (0.76, 1.04)		0.87 (0.74, 1.03)		0.88 (0.79, 0.99)	
Race/ethnicity						
Non‐hispanic white/Han	0.86 (0.76, 0.98)	0.09	0.95 (0.87, 1.04)	0.20	0.92 (0.85, 0.99)	0.21
Other	0.89 (0.76, 1.05)		0.79 (0.53, 1.19)		0.88 (0.75, 1.02)	
Education level						
Less than high school graduate	0.88 (0.79, 0.98)	0.41	0.91 (0.82, 1.01)	0.21	0.90 (0.83, 0.97)	0.21
High school graduate or above	0.85 (0.74, 0.98)		1.17 (0.91, 1.51)		0.92 (0.81, 1.05)	
BMI^b^						
<Median	0.87 (0.73, 1.03)	0.98	0.93 (0.84, 1.03)	0.38	0.94 (0.87, 1.00)	0.40
≥Median	0.87 (0.76, 0.98)		0.93 (0.77, 1.12)		0.89 (0.81, 0.97)	
Economic status						
Low	0.88 (0.73, 1.06)	0.73	0.91 (0.81, 1.01)	0.68	0.90 (0.82, 0.99)	0.75
High	0.87 (0.76, 0.98)		0.97 (0.85, 1.11)		0.92 (0.84, 1.00)	
Drinking status						
Nondrinker	0.85 (0.75, 0.96)	0.77	0.91 (0.82, 1.01)	0.12	0.88 (0.82, 0.96)	0.49
Drinker	0.87 (0.75, 1.00)		1.18 (0.91, 1.53)		0.93 (0.82, 1.06)	
Smoking status						
Never	0.91 (0.78, 1.05)	0.96	0.91 (0.81, 1.03)	0.93	0.91 (0.83, 1.00)	0.83
Ever or current	0.84 (0.73, 0.97)		0.99 (0.85, 1.15)		0.94 (0.83, 1.06)	
Leisure‐time physical activity						
Inactive	0.83 (0.68, 1.03)	0.77	1.03 (0.86, 1.24)	0.75	0.94 (0.82, 1.08)	0.61
Active	0.88 (0.78, 0.99)		0.93 (0.84, 1.04)		0.91 (0.84, 0.98)	
Medication for diabetes						
No	0.98 (0.78, 1.23)	0.95	1.03 (0.87, 1.23)	0.28	1.01 (0.88, 1.16)	0.96
Yes	0.86 (0.77, 0.96)		0.89 (0.79, 1.00)		0.87 (0.81, 0.95)	
Diabetes duration, y						
< 3	0.96 (0.83, 1.10)	0.66	0.94 (0.84, 1.04)	0.93	0.95 (0.87, 1.03)	0.93
≥ 3	0.81 (0.70, 0.93)		0.99 (0.83, 1.18)		0.87 (0.78, 0.97)	
Hypertension and/or Hypercholesterolemia						
Yes	0.83 (0.74, 0.94)	0.90	0.94 (0.83, 1.07)	0.74	0.88 (0.81, 0.96)	0.61
No	0.97 (0.79, 1.19)		0.94 (0.82, 1.06)		0.95 (0.85, 1.06)	
CVD and/or cancer						
Yes	0.95 (0.83, 1.08)	0.05	0.89 (0.75, 1.05)	0.82	0.92 (0.83, 1.02)	0.21
No	0.80 (0.70, 0.91)		0.95 (0.84, 1.07)		0.88 (0.80, 0.96)	
Marine n3 PUFA						
<Median	0.78 (0.68, 0.89)	0.07	0.94 (0.84, 1.07)	0.58	0.86 (0.79, 0.95)	0.34
≥Median	0.98 (0.85, 1.14)		0.95 (0.81, 1.11)		0.97 (0.87, 1.08)	

*Note:* HRs were adjusted for age, sex, race/ethnicity, BMI, education level, economic status, drinking status, smoking status, leisure‐time physical activity, history of hypertension, history of hypercholesterolemia, history of cancer, history of cardiovascular diseases, family history of cardiovascular diseases, family history of diabetes, duration of diabetes, diabetes medication use, ALA supplement, and total energy intake. The strata variable was not included when stratifying by itself.

Abbreviations: ALA, alpha‐linolenic acid; CHNS, China health and nutrition survey; CVD, cardiovascular disease; NHANES, national health and nutrition examination survey.

^a^ Results for NHANES and CHNS from the multivariable model were combined using the fixed‐effects model.

^b^ NHANES: < 30 or ≥ 30; CHNS: < 25 or ≥ 25.

### 3.3 Cardiometabolic Biomarkers

The least‐squares means of cardiometabolic biomarkers based on ALA intake were shown in Table S5. Higher ALA intake was significantly associated with lower levels of TC in both cohorts at baseline. In addition, ALA intake demonstrated an inverse association with TG levels in NHANES and LDL‐C in CHNS, while exhibiting a positive association with TG levels in CHNS (all *p*_trend_ < 0.05).

## 4 Discussion

Leveraging two nationally representative populations of China and the US, this study explored the association between ALA intake and mortality among participants with T2D. Our findings revealed an inverse association between higher dietary ALA intake and all‐cause as well as CVD mortality, exhibiting a linear dose–response pattern among adults with T2D. Consistent findings were observed in both stratified and sensitivity analyses. These findings emphasized the significance of ALA intake in reducing the mortality risk among adults with T2D.

Previous results from NHS and HPFS indicated a marginally reverse association between ALA intake in comparison with SFAs and all‐cause mortality among adults with T2D [14]. Likewise, in the current study involving populations with T2D, each 1 g/day increment in ALA intake was linked to a 9% lower risk of all‐cause mortality upon combining the results from NHANES and CHNS. It is noteworthy that the reverse association was more prominent in NHANES than CHNS. Despite the disparity in sample size (2301 deaths in NHANES and 167 deaths in CHNS), the median ALA intake was higher among Chinese populations than US populations (1.54 vs. 1.25 g/day), potentially accounting for the varied results. A meta‐analysis of prospective studies indicated that a moderate ALA intake was associated with a decreased incidence rate of T2D [20]. In the current study, the risk of mortality was observed to significantly decrease up to 2.5 g/day, with the trend gradually diminishing with higher ALA intake. This level of ALA intake exceeds current general population guidelines (1.1 g/day for women and 1.6 g/day for men in the US; 0.6% energy per day in China, about 1.4–1.7 g/day), underscoring the potential need for ALA recommendations in T2D management. Our findings suggest that individuals with T2D may benefit from ALA intakes exceeding current general population guidelines, especially from ALA‐rich foods, such as one spoonful of flaxseed oil (about 4 g), one spoonful of chia seeds (about 15 g), 5–6 walnuts (about 40 g), or consider supplements under clinical guidance. Of course, further studies are needed to establish optimal dosage of ALA intake for populations with T2D, while accounting for regional dietary contexts.

In addition, higher dietary ALA intake was associated with a lower risk of CVD mortality among US adults with T2D, which was consistent with earlier epidemiological evidence [24, 25]. Prior meta‐analyses of prospective studies [26] and randomized clinical trials [13] have indicated that increasing ALA intake provided cardiovascular benefits in the general population. However, no significant association was observed between increased ALA intake and CVD mortality among adults with cardiometabolic disease [27]. Thus, further studies are needed to investigate the relationships among individuals with various chronic diseases.

Our findings indicated that increased ALA intake was linked to decreased TC levels in both cohorts, reduced TG levels in NHANES, and reduced LDL‐C levels in CHNS. These findings were in accordance with previous randomized controlled trials, demonstrating the beneficial effects of ALA on glycolipid metabolism among populations with T2D [28, 29]. Although our findings about LDL‐C and TG were inconsistent, which noted a slight increase in LDL‐C level among individuals in NHANES and an increased TG level among individuals in CHNS, it may be partially due to the different food sources of ALA and the genetic backgrounds of the two populations. The data of this study showed that individuals with T2D mainly obtained ALA from vegetable oils (76.2%), vegetables, and legumes (10.5%) in CHNS, while from animal‐based foods (32.5%, such as red meat, poultry, eggs and dairy products, etc.), grains (23.9%), vegetables, and legumes (17.3%) in NHANES. The Chinese population mainly consumes rapeseed oil and soybean oil, which contain rich PUFA and MUFA as well as less SFA. On the contrary, animal‐based foods contain much SFA, while PUFA and MUFA are relatively low. Studies suggested that excessive intake of SFA may increase the burden of blood lipids and lead to an increase in LDL‐C, whereas plant‐derived PUFA/MUFA could improve lipid homeostasis [30, 31, 32]. And there is evidence showing that apolipoprotein E (ApoE) polymorphism could affect plasma lipid traits, and the frequency of ApoE alleles differed significantly across ethnic groups [33, 34]. In addition, a most recent meta‐analysis of randomized controlled trials suggested that ALA intake could improve serum TC and LDL‐C levels among subjects with hyperglycemia [35], potentially leading to a decreased risk of CVD and mortality.

There are several potential mechanisms underlying the association between ALA intake and mortality among individuals with T2D. First, ALA intake may promote the synthesis and release of insulin, as well as improve insulin sensitivity [36, 37]. Second, an ALA‐enriched diet was observed to regulate metabolism‐related genes and inhibit the expression of inflammatory factors to exert potential cardiovascular benefits [38, 39]. Thirdly, although a small proportion of ALA could be converted into EPA and DHA, cumulative accretion of EPA/DHA could enhance anti‐inflammatory levels and minimize the risk of complications and mortality among individuals with T2D [40, 41].

Strengths of our study included the use of nationally representative samples of Chinese and US populations with T2D. In various sensitivity analyses, the majority of documented associations remained unchanged, demonstrating the robustness of our results. However, this study had certain limitations. First, the dietary information relied on 24‐h dietary recalls at baseline, which may potentially introduce recall bias and might not capture changes in intake over time. In addition, including participants from 1999 to 2002 (with data from only one dietary review) might overestimate or underestimate their dietary ALA intake and introduce new confounding. Second, an oral glucose tolerance test (OGTT) was solely accessible in the cycles from 2005 to 2016 in NHANES, and data on HbA1c was only available in the cycle in 2009 in CHNS, which likely resulted in an underestimation of the number of adults with T2D. Third, there were some differences between CHNS and NHANES in multivariate adjustment due to CHNS lacking information about the family history of the disease and fatty acid supplements. What's more, since the data on the specific causes of death in CHNS cannot be obtained, the results regarding CVD mortality are completely based on NHANES, which will limit the expansion of this result to other populations. Fourth, given the nature of observational studies, reverse causation could potentially occur. Thus, we excluded the participants with cancer or extreme BMI at baseline and those who died within the first 4 years since baseline, observing consistent results. Last, the potential for unmeasured confounding variables was inevitably considering the observational nature of the study, despite our adjustments for demographic, socioeconomic, lifestyle, and medical history variables. However, we computed *E*‐values, indicating that unmeasured confounders were likely not able to offset the main associations.

## 5 Conclusions

In the two nationally representative cohort studies of US and Chinese populations, higher dietary ALA intake is significantly associated with a lower risk of all‐cause and CVD mortality among adults with T2D. This indicates that increasing ALA intake may help mitigate the risk of premature mortality in this population. If confirmed by further prospective studies, these findings could inform prevention strategies targeting dietary recommendations to promote higher ALA intake among adults with T2D.

## Author Contributions

Z.S. and J.Y. conceived and designed the study. B.C. and Q.W. did the statistical analysis. B.C. and Q.W. drafted the article. Z.S., J.Y., and L.L. critically revised the article. Z.S. and J.Y. supervised the study and provided administrative, technical, and material support. Z.S. and J.Y. had full access to all the data in the study and took responsibility for the integrity of the data and the accuracy of the data analysis. All authors acquired, analyzed, and interpreted the data or critically revised the article for important intellectual content. All authors have read and approved the final version.

## Conflicts of Interest

The authors declare no conflicts of interest.

## Supporting information


**Table S1.** Associations between alpha‐linolenic acid intake with cardiovascular disease mortality among adults with type 2 diabetes.
**Table S2.** Subgroup analyses of associations between alpha‐linolenic acid intake and cardiovascular disease mortality among adults with type 2 diabetes in NHANES.
**Table S3.** Sensitivity analyses of associations with alpha‐linolenic acid intake and all‐cause mortality among adults with type 2 diabetes.
**Table S4.** Sensitivity analyses of associations with alpha‐linolenic acid intake and cardiovascular disease mortality among adults with type 2 diabetes.
**Table S5.** Least squares mean of cardiometabolic markers according to alpha‐linolenic acid intake among participants with diabetes in NHANES 1999–2018 and CHNS 1997–2011.
**Figure S1.** The flowchart of the study population.
**Figure S2.** The dose–response relation between alpha‐linolenic acid intake and cardiovascular disease mortality with restricted cubic spline regression model in NHANES.
