# Supplementary material for: Alpha‐Linolenic Acid and Mortality Among Adults With Type 2 Diabetes: Findings From Two National Cohorts
Source: J Diabetes. 2025 Jun 22;17(6):e70110. doi: 10.1111/1753-0407.70110 (PMC12183326; doi:10.1111/1753-0407.70110)
Supplement: Supplementary file 1 — Table S1. Associations between alpha‐linolenic acid intake with cardiovascular disease mortality among adults with type 2 diabetes. Table S2. Subgroup analyses of associations between alpha‐linolenic acid intake and cardiovascular disease mortality among adults with type 2 diabetes in NHANES. Table S3. Sensitivity analyses of associations with alpha‐linolenic acid intake and all‐cause mortality among adults with type 2 diabetes. Table S4. Sensitivity analyses of associations with alpha‐linolenic acid intake and cardiovascular disease mortality among adults with type 2 diabetes. Table S5. Least squares mean of cardiometabolic markers according to alpha‐linolenic acid intake among participants with diabetes in NHANES 1999–2018 and CHNS 1997–2011. Figure S1. The flowchart of the study population. Figure S2. The dose–response relation between alpha‐linolenic acid intake and cardiovascular disease mortality with restricted cubic spline regression model in NHANES. [file JDB-17-e70110-s001.docx]

**Supplemental Material**

**Supplementary Table 1.** Associations between alpha-linolenic acid intake with cardiovascular disease mortality among adults with type 2 diabetes.

**Supplementary Table 2.** Subgroup analyses of associations between alpha-linolenic acid intake and cardiovascular disease mortality among adults with type 2 diabetes in NHANES.

**Supplementary Table 3.** Sensitivity analyses of associations with alpha-linolenic acid intake and all-cause mortality among adults with type 2 diabetes.

**Supplementary Table 4.** Sensitivity analyses of associations with alpha-linolenic acid intake and cardiovascular disease mortality among adults with type 2 diabetes.

**Supplementary Table 5.** Least squares mean of cardiometabolic markers according to alpha-linolenic acid intake among participants with diabetes in NHANES 1999–2018 and CHNS 1997-2011.

**Supplementary Fig 1.** The flowchart of the study population.

**Supplementary Fig 2.** The dose-response relation between alpha-linolenic acid intake and cardiovascular disease mortality with restricted cubic spline regression model in NHANES.

**Supplementary Table 1.** Associations between alpha-linolenic acid intake with cardiovascular disease mortality among adults with type 2 diabetes in NHANES*.

|  | **Tertiles of ALA intake** | | | **Per 1 g/day increase** | ***P trend*** |
| --- | --- | --- | --- | --- | --- |
|  | **T1**  **0.68 (<1.01)** | **T2**  **1.25 (1.01-1.67)** | **T3**  **2.17 (>1.67)** |  |  |
| No. of deaths/person-years | 344/22,559 | 258/20,954 | 178/19,640 |  |  |
| Model 1^†^ | 1.00 | 0.77 (0.61,0.96) | 0.58 (0.45,0.76) | 0.81 (0.68,0.96) | <0.01 |
| Model 2^‡^ | 1.00 | 0.81 (0.65,1.01) | 0.68 (0.50,0.91) | 0.90 (0.74,1.11) | 0.01 |

Abbreviations: ALA, alpha-linolenic acid; NHANES, National Health and Nutrition Examination Survey.

***** The analysis was performed only in NHANES.

† Adjusted for age (continuous), sex (male or female), and race/ ethnicity (non-Hispanic white, non-Hispanic Black, Mexican American, or other).

‡ Further adjusted for BMI (<18.5, 18.5-24.9, 25.0-29.9, or ≥30.0), education level (less than high school, high school or equivalent, or college or above), economy status (ratio of family income poverty, ≤1.0, 1.0-3.0, or >3.0), drinking status (non-drinker, ever drinker, low-to-moderate drinker, or heavy drinker), smoking status (never smoker, ever smoker, or current smoker, <20 or ≥20 cigarettes per day), leisure-time physical activity (inactive, insufficiently active, active), history of hypertension (yes, no), history of hypercholesterolemia (yes, no), history of cancer (yes, no), history of cardiovascular diseases (yes, no), family history of cardiovascular diseases (yes, no), family history of diabetes (yes, no), duration of diabetes (≤3, 3–10, or >10 years), diabetes medication use (none, oral medication and/or insulin), ALA supplement (yes, no), and total energy intake (continuous).

**Supplementary Table 2.** Subgroup analyses of associations between alpha-linolenic acid intake and cardiovascular disease mortality among adults with type 2 diabetes in NHANES*.

|  | **Marine n3 PUFA**^†^ | | ***P* _interaction_** |
| --- | --- | --- | --- |
|  | **< Median** | **≥ Median** |  |
| Per 1 g/day increase^‡^ | 0.66 (0.50,0.85) | 1.15 (0.92,1.45) | 0.06 |

***** The analysis was conducted only in NHANES.

† The median intake of marine n3 PUFA was 0.05g/day.

‡ HR were adjusted for age (continuous), sex (male or female), and race/ ethnicity (non-Hispanic white, non-Hispanic Black, Mexican American, or other), BMI (<18.5, 18.5-24.9, 25.0-29.9, or ≥30.0), education level (less than high school, high school or equivalent, or college or above), economy status (ratio of family income poverty, ≤1.0, 1.0-3.0, or >3.0), drinking status (non-drinker, ever drinker, low-to-moderate drinker, or heavy drinker), smoking status (never smoker, ever smoker, or current smoker, <20 or ≥20 cigarettes per day), leisure-time physical activity (inactive, insufficiently active, active), history of hypertension (yes, no), history of hypercholesterolemia (yes, no), history of cancer (yes, no), history of cardiovascular diseases (yes, no), family history of cardiovascular diseases (yes, no), family history of diabetes (yes, no), duration of diabetes (≤3, 3–10, or >10 years), diabetes medication use (none, oral medication and/or insulin), ALA supplement (yes, no), and total energy intake (continuous).

**Supplementary Table 3.** Sensitivity analyses of associations with alpha-linolenic acid intake and all-cause mortality among adults with type 2 diabetes*****.

|  | **Tertiles of ALA intake** | | | **Per 1 g/day increase** | ***P trend*** |
| --- | --- | --- | --- | --- | --- |
|  | **T 1 (lowest)** | **T 2 (medium)** | **T 3 (highest)** |  |  |
| **Excluding participants with CVD** | | | | | |
| **NHANES** | 1.00 | 0.88 (0.74,1.03) | 0.77 (0.63,0.96) | 0.87 (0.78,0.96) | 0.02 |
| **CHNS** | 1.00 | 0.60 (0.36,1.00) | 0.73 (0.42,1.27) | 0.96 (0.85,1.08) | 0.29 |
| **Pooled** | 1.00 | 0.85 (0.73,0.99) | 0.76 (0.63,0.93) | 0.91 (0.84,0.98) | 0.01 |
| **Excluding participants with cancer** | | | | | |
| **NHANES** | 1.00 | 0.87 (0.74,1.03) | 0.78 (0.63,0.96) | 0.87 (0.78,0.96) | 0.02 |
| **CHNS** | 1.00 | 0.72 (0.49,1.06) | 0.75 (0.50,1.11) | 0.94 (0.86,1.03) | 0.19 |
| **Pooled** | 1.00 | 0.84 (0.73,0.98) | 0.77 (0.64,0.93) | 0.93 (0.89,0.97) | 0.01 |
| **Excluding deaths within the first 4 years since baseline**^†^ | | | | | |
| **NHANES** | 1.00 | 0.91 (0.77,1.08) | 0.78 (0.62,0.99) | 0.87 (0.77,0.98) | 0.04 |
| **CHNS** | 1.00 | 0.70 (0.39,1.24) | 0.66 (0.37,1.21) | 0.91 (0.79,1.05) | 0.22 |
| **Pooled** | 1.00 | 0.89 (0.76,1.05) | 0.76 (0.61,0.95) | 0.93 (0.88,0.98) | 0.01 |
| **Excluding participants in 1999-2002 in NHANES** | | | | | |
| **NHANES** | 1.00 | 0.92 (0.78,1.09) | 0.85 (0.68,1.05) | 0.86 (0.76,0.98) | 0.13 |
| **CHNS** | 1.00 | 0.72 (0.49,1.06) | 0.75 (0.50,1.11) | 0.94 (0.86,1.03) | 0.19 |
| **Pooled** | 1.00 | 0.88 (0.76,1.03) | 0.83 (0.68,1.00) | 0.91 (0.85,0.98) | 0.04 |
| **Excluding extreme BMI**^‡^ | | | | | |
| **NHANES** | 1.00 | 0.86 (0.73,1.00) | 0.79 (0.65,0.97) | 0.86 (0.78,0.95) | 0.03 |
| **CHNS** | 1.00 | 0.74 (0.48,1.14) | 0.71 (0.45,1.11) | 0.93 (0.84,1.03) | 0.16 |
| **Pooled** | 1.00 | 0.84 (0.73,0.97) | 0.78 (0.65,0.93) | 0.92 (0.88,0.96) | 0.01 |
| **Converting ALA unit to %E** | | | | | |
| **NHANES** | 1.00 | 1.04 (0.90,1.21) | 0.85 (0.74,0.99) | 0.79 (0.66,0.95) | 0.02 |
| **CHNS** | 1.00 | 0.73 (0.50,1.07) | 0.69 (0.47,1.00) | 0.86 (0.69,1.06) | 0.07 |
| **Pooled** | 1.00 | 0.99 (0.86,1.14) | 0.83 (0.72,0.95) | 0.82 (0.71,0.94) | 0.01 |
| **Multiple imputation** | | | | | |
| **NHANES** | 1.00 | 0.90 (0.78,1.03) | 0.78 (0.65,0.94) | 0.86 (0.78,0.95) | 0.01 |
| **CHNS** | 1.00 | 0.72 (0.49,1.06) | 0.75 (0.50,1.12) | 0.94 (0.86,1.03) | 0.19 |
| **Pooled** | 1.00 | 0.88 (0.77,1.00) | 0.77 (0.66,0.92) | 0.90 (0.84,0.96) | 0.01 |

Abbreviations: ALA, alpha-linolenic acid; CHNS, China Health and Nutrition Survey; NHANES, National Health and Nutrition Examination Survey.

***** HRs were adjusted for age (continuous), sex (male or female), and race/ ethnicity (NHANES: non-Hispanic white, non-Hispanic Black, Mexican American, or other; CHNS: Han, the minority), BMI (calculated as weight in kilograms divided by height in meters squared; NHANES: <18.5, 18.5-24.9, 25.0-29.9, or ≥30.0; CHNS: <18.5, 18.5-23.9, 24.0-27.9, or ≥28.0), education level (less than high school, high school or equivalent, or college or above), economy status (NHANES: ratio of family income poverty, ≤1.0, 1.0-3.0,or >3.0; CHNS: per capita annual household income, ¥, <5000, 5000-10000, or >10000), drinking status (non-drinker, ever drinker, low-to-moderate drinker, or heavy drinker), smoking status (never smoker, ever smoker, or current smoker, <20 or ≥20 cigarettes per day), leisure-time moderate-to-vigorous physical activity (inactive, insufficiently active, active), history of hypertension (yes, no), history of hypercholesterolemia (yes, no), history of cardiovascular diseases (yes, no), family history of cardiovascular diseases (yes, no, only for NHANES), family history of diabetes (yes, no, only for NHANES), duration of diabetes (≤3, 3–10, or >10 years), diabetes medication use (none, oral medication and/or insulin), ALA supplement (yes, no, only for NHANES), and total energy intake (continuous).

† Further adjusted for history of cancer (yes, no).

‡ Extreme BMI: <18.5 or >40 kg/m^2^.

**Supplementary Table 4**. Sensitivity analyses of associations with alpha-linolenic acid intake and cardiovascular disease mortality among adults with type 2 diabetes in NHANES.

|  | **Tertiles of ALA intake** | | | **Per 1 g/day increase** | ***P trend*** |
| --- | --- | --- | --- | --- | --- |
|  | **T 1 (lowest)** | **T 2 (medium)** | **T 3 (highest)** |  |  |
| **Excluding participants with CVD** | 1.00 | 0.82 (0.63,1.06) | 0.58 (0.42,0.81) | 0.92 (0.72,1.17) | <0.01 |
| **Excluding participants with cancer** | 1.00 | 0.81 (0.63,1.05) | 0.60 (0.43,0.83) | 0.92 (0.72,1.17) | <0.01 |
| **Excluding deaths within first 4 years since baseline**^†^ | 1.00 | 0.94 (0.73,1.22) | 0.69 (0.48,1.00) | 0.98 (0.76,1.26) | 0.04 |
| **Excluding extreme BMI**^‡^ | 1.00 | 0.80 (0.62,1.02) | 0.62 (0.46,0.85) | 0.85 (0.69,1.04) | <0.01 |
| **Converting ALA unit to %E** | 1.00 | 1.14 (0.88,1.46) | 0.77 (0.59,1.00) | 0.79 (0.54,1.15) | 0.03 |
| **Multiple imputation** | 1.00 | 0.82 (0.66,1.01) | 0.66 (0.49,0.88) | 0.89 (0.72,1.09) | 0.01 |

Abbreviations: ALA, alpha-linolenic acid; NHANES, National Health and Nutrition Examination Survey.

***** HRs were adjusted for age (continuous), sex (male or female), and race/ ethnicity (NHANES: non-Hispanic white, non-Hispanic Black, Mexican American, or other), BMI (calculated as weight in kilograms divided by height in meters squared; NHANES: <18.5, 18.5-24.9, 25.0-29.9, or ≥30.0), education level (less than high school, high school or equivalent, or college or above), economy status (NHANES: ratio of family income poverty, ≤1.0, 1.0-3.0,or >3.0), drinking status (non-drinker, ever drinker, low-to-moderate drinker, or heavy drinker), smoking status (never smoker, ever smoker, or current smoker, <20 or ≥20 cigarettes per day), leisure-time moderate-to-vigorous physical activity (inactive, insufficiently active, active), history of hypertension (yes, no), history of hypercholesterolemia (yes, no), history of cardiovascular diseases (yes, no), family history of cardiovascular diseases (yes, no), family history of diabetes (yes, no), duration of diabetes (≤3, 3–10, or >10 years), diabetes medication use (none, oral medication and/or insulin), ALA supplement (yes, no), and total energy intake (continuous). This analysis was performed only in NHANES.

† Further adjusted for history of cancer (yes, no).

‡ Extreme BMI: <18.5 or >40 kg/m^2^.

**Supplementary Table 5**. Least squares mean of cardiometabolic markers according to alpha-linolenic acid intake among participants with diabetes in NHANES 1999–2018 and CHNS 1997-2011.

|  | **Tertiles of ALA intake** | | | ***P trend*** |
| --- | --- | --- | --- | --- |
|  | **T1 (lowest)** | **T2 (medium)** | **T3 (highest)** |  |
| **NHANES** |  |  |  |  |
| ALA intake, g/day | 0.68 (<1.01) | 1.25 (1.01-1.67) | 2.17 (>1.67) |  |
| Glucose (n=625), mmol/L | 8.32±1.10 | 8.55±1.10 | 8.43±1.11 | 0.51 |
| Insulin (n=641), pmol/L | 115.13±32.19 | 124.91±32.05 | 121.48±32.53 | 0.94 |
| HOMA2-IR (n=610) | 2.37±0.66 | 2.57±0.65 | 2.60±0.66 | 0.49 |
| HbA1c (n=1,185), % (mmol/mol) | 6.06±0.41 | 6.12±0.41 | 6.16±0.41 | 0.53 |
| Total cholesterol (n=1,163), mmol/L | 5.43±0.32 | 5.43±0.32 | 5.19±0.32 | **0.02** |
| HDL-C (n=1,163), mmol/L | 1.30±0.09 | 1.30±0.09 | 1.32±0.09 | 0.21 |
| LDL-C (n=582), mmol/L | 2.97±0.36 | 3.09±0.36 | 2.98±0.36 | 0.44 |
| Triglyceride (n=608), mmol/L | 2.58±0.51 | 2.37±0.51 | 2.24±0.52 | **0.02** |
| CRP (n=532), mg/L | 8.43±4.28 | 6.56±4.33 | 7.89±4.32 | 0.61 |
| **CHNS** |  |  |  |  |
| ALA intake, g/day | 0.33 (<0.81) | 1.54 (0.81-2.37) | 3.58 (>2.37) |  |
| Glucose (n=565), mmol/L | 7.49±0.96 | 7.47±0.95 | 7.44±0.96 | 0.86 |
| Insulin (n=563), pmol/L | 252.24±87.01 | 246.74±86.32 | 241.12±87.60 | 0.67 |
| HOMA2-IR (n=559) | 4.25±1.43 | 4.05±1.42 | 4.12±1.44 | 0.80 |
| HbA1c (n=564), % (mmol/mol) | 6.11±0.69 | 6.32±0.68 | 6.31±0.69 | 0.36 |
| Total cholesterol (n=565), mmol/L | 5.14±0.36 | 4.90±0.36 | 4.83±0.36 | **0.005** |
| HDL-C (n=564), mmol/L | 1.38±0.18 | 1.41±0.18 | 1.32±0.18 | 0.19 |
| LDL-C (n=564), mmol/L | 3.06±0.41 | 2.77±0.40 | 2.56±0.41 | **<0.01** |
| Triglyceride (n=565), mmol/L | 2.25±0.81 | 2.43±0.80 | 2.82±0.81 | **0.02** |
| CRP (n=564), mg/L | 5.78±5.47 | 4.48±5.43 | 4.33±5.50 | 0.40 |

Abbreviations: ALA, alpha-linolenic acid; CHNS, China Health and Nutrition Survey; CRP, C-reactive protein; HbA1c, glycated hemoglobin; HDL-C, high-density lipoprotein cholesterol; HOMA2-IR, homoeostasis model assessment (HOMA) 2 estimates of insulin resistance; LDL-C, low-density lipoprotein cholesterol; NHANES, National Health and Nutrition Examination Survey.

Values are mean ± SE unless otherwise indicated. The least square estimated using general linear model with adjustment of age (continuous), sex (male or female), and race/ ethnicity (NHANES: non-Hispanic white, non-Hispanic Black, Mexican American, or other; CHNS: Han, the minority), BMI (calculated as weight in kilograms divided by height in meters squared; NHANES: <18.5, 18.5-24.9, 25.0-29.9, or ≥30.0; CHNS: <18.5, 18.5-23.9, 24.0-27.9, or ≥28.0), education level (less than high school, high school or equivalent, or college or above), economy status (NHANES: ratio of family income poverty, ≤1.0, 1.0-3.0,or >3.0; CHNS: per capita annual household income, ¥, <5000, 5000-10000, or >10000), drinking status (non-drinker, ever drinker, low-to-moderate drinker, or heavy drinker), smoking status (never smoker, ever smoker, or current smoker, <20 or ≥20 cigarettes per day), leisure-time moderate-to-vigorous physical activity (inactive, insufficiently active, active), history of hypertension (yes, no), history of hypercholesterolemia (yes, no), history of cancer (yes, no), history of cardiovascular diseases (yes, no), family history of cardiovascular diseases (yes, no, only for NHANES), family history of diabetes (yes, no, only for NHANES), duration of diabetes (≤3, 3–10, or >10 years), diabetes medication use (none, oral medication and/or insulin), ALA supplement (yes, no, only for NHANES), and total energy intake (continuous).

**
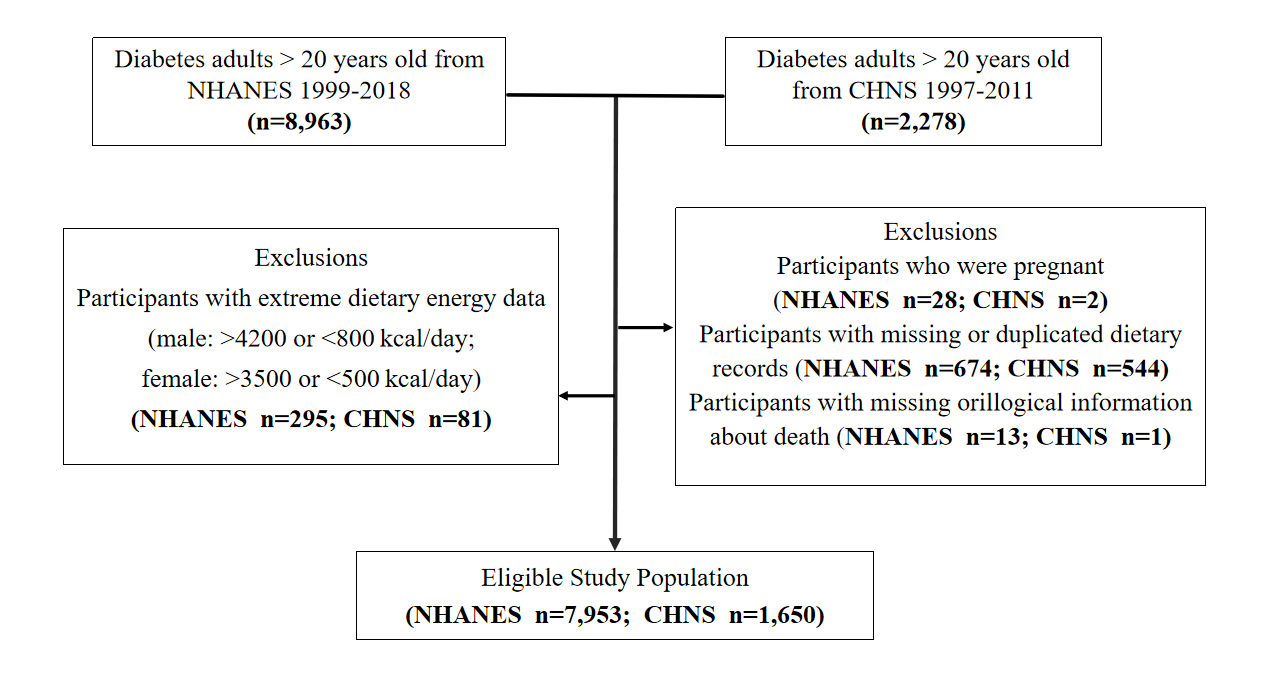
**

**Supplementary Fig. 1.** The flowchart of the study population.


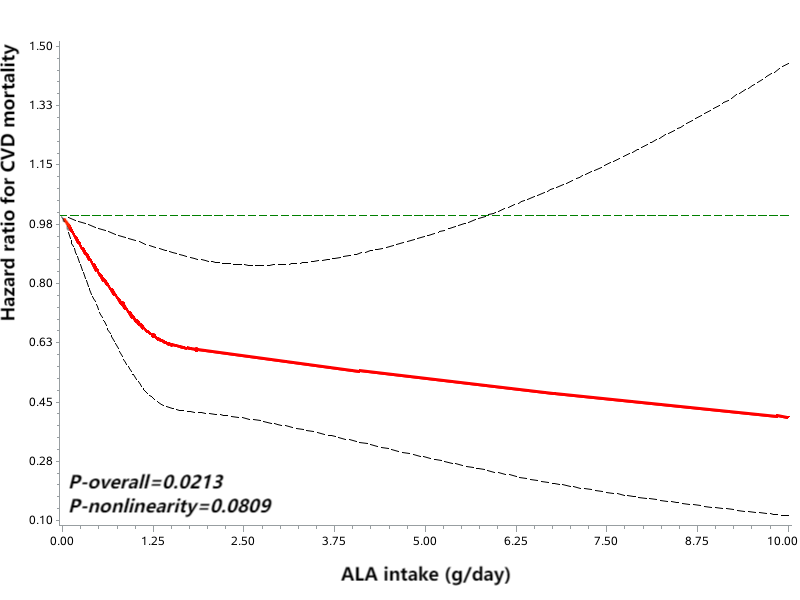


**Supplementary Fig 2.** The dose-response relation between alpha-linolenic acid intake and cardiovascular disease mortality with restricted cubic spline regression model in NHANES.

Abbreviations: ALA, alpha-linolenic acid; NHANES, National Health and Nutrition Examination Survey.

HRs were adjusted for age (continuous), sex (male or female), and race/ ethnicity (non-Hispanic white, non-Hispanic Black, Mexican American, or other), BMI (calculated as weight in kilograms divided by height in meters squared; <18.5, 18.5-24.9, 25.0-29.9, or ≥30.0), education level (less than high school, high school or equivalent, or college or above), economy status ( ratio of family income poverty, ≤1.0, 1.0-3.0, or >3.0), drinking status (non-drinker, ever drinker, low-to-moderate drinker, or heavy drinker), smoking status (never smoker, ever smoker, or current smoker, <20 or ≥20 cigarettes per day), leisure-time moderate-to-vigorous physical activity (inactive, insufficiently active, active), history of hypertension (yes, no), history of hypercholesterolemia (yes, no), history of cancer (yes, no), history of cardiovascular diseases (yes, no), family history of cardiovascular diseases (yes, no), family history of diabetes (yes, no), duration of diabetes (≤3, 3-10, or >10 years), diabetes medication use (none, oral medication and/or insulin), ALA supplement (yes, no), and total energy intake (continuous).
